# Trends in Use of Biomarker Protocols for the Evaluation of Possible Myocardial Infarction

**DOI:** 10.1161/JAHA.117.005852

**Published:** 2017-09-22

**Authors:** Brian J. Hachey, Michael C. Kontos, L. Kristin Newby, Robert H. Christenson, W. Frank Peacock, Katherine C. Brewer, James McCord

**Affiliations:** ^1^ Department of Cardiology Advocate Illinois Masonic Medical Center Chicago IL; ^2^ Pauley Heart Center Virginia Commonwealth University Richmond VA; ^3^ Department of Cardiology Duke University Durham NC; ^4^ Department of Pathology University of Maryland School of Medicine Baltimore MD; ^5^ Department of Emergency Medicine Baylor College of Medicine Houston TX; ^6^ Department of Research Advocate Illinois Masonic Medical Center Chicago IL; ^7^ Department of Cardiology Henry Ford Hospital Detroit MI

**Keywords:** cardiac biomarkers, myocardial infarction, trends, troponin, Biomarkers, Myocardial Infarction, Cost-Effectiveness

## Abstract

**Background:**

Various combinations of creatine kinase‐MB, myoglobin, and cardiac troponin I or T (cTnI/cTnT) have been used to evaluate patients with suspected acute coronary syndromes. The current recommendation is to use the 99th percentile of cTnI/cTnT as the sole marker for diagnosis of acute myocardial infarction.

**Methods and Results:**

We retrospectively analyzed cardiac marker protocols collected from 824 US hospitals undergoing Chest Pain Center Accreditation through the Society of Cardiovascular Patient Care from 2009 to 2014. Data were obtained by a self‐reported survey that addressed cardiac marker(s), sampling time periods, and cut points used for evaluation of suspected acute myocardial infarction. The combination of cTnI or cTnT with creatine kinase‐MB was the most commonly used biomarker strategy. Use of cTnI or cTnT as the sole marker increased over time (14–37%; *P*<0.0001), as did use of the 99th percentile cut point for cTnI/cTnT (30–60%; *P*<0.0001).

**Conclusion:**

There is considerable variation in cardiac marker testing strategies used in US hospitals for evaluation of suspected acute myocardial infarction. Although increasing, 24% of hospitals used a cTn alone strategy, and only 49% used cTn at the recommended 99th percentile cut point. This has important implications for the diagnosis and treatment of patients with acute myocardial infarction.

**Clinical Perspective**

**What Is New?**

- In the rule‐out acute myocardial infarction process, cardiac troponin is the sole recommended biomarker, using the 99th percentile cut point and with 2 lab draws at time 0 and 3 to 6 hours.
- When comparing US hospitals to guidelines, only 24% used a cardiac troponin–only strategy, 49% used the 99th percentile cut point, and 35% ruled out myocardial infarction within 6 hours.

**What Are the Clinical Implications?**

- Use of cardiac biomarkers in the acute myocardial infarction rule‐out process that is not consistent with recommended guidelines leads to increased cost and longer acute myocardial infarction rule‐out times without clinical benefit.

Early diagnosis of acute myocardial infarction (AMI) relies on the history, ECG, and cardiac biomarkers. Because clinical history and physical examination alone have limited utility,^1^ and a 12‐lead ECG is diagnostic in a minority of cases, cardiac biomarkers are the cornerstone for diagnosis of non‐ST‐segment elevation myocardial infarction.^2^

Over the past 20 years, cardiac troponin (cTn), creatine kinase (CK)‐MB, and myoglobin have been the most commonly used cardiac biomarkers to identify myocardial necrosis in the evaluation of patients with suspected AMI. Several strategies have been evaluated, including the use of biomarker combinations, sampling at various time intervals, and use of variable cut points. Because of its high sensitivity and specificity for myocardial injury, cTn is the gold‐standard biomarker for risk stratification and diagnosis of AMI. Since the initial use of cTn, the sensitivity and precision of cTn assays have significantly improved, such that use of other markers is no longer recommended.^2^

The second universal definition of myocardial infarction consensus document,^3^ published in 2007, recommended specific criteria for diagnosis of AMI, in which cTnI or cTnT was the preferred cardiac biomarker with sample measurement at time 0 (presentation), followed by repeat sampling 6 to 9 hours later, and included a rise or fall of cTn with at least 1 value exceeding the 99th percentile reference range cut point for AMI diagnosis, using a recommended optimal assay precision at the 99th percentile of <10% coefficient of variation.^3^ Importantly, use of the 99th percentile as the cut‐off value increases the frequency of AMI diagnosis, has been associated with improved outcomes, and is championed as a Laboratory Medicine Best Practice.^4^, ^5^, ^6^ Guidelines were updated in 2012, shortening the marker sampling interval to 3 to 6 hours, reflecting the improved cTn assay sensitivity.^3^ In this analysis, we assessed biomarker strategies used by US hospitals to evaluate suspected AMI patients, identified changes in strategy over time, and compared findings with the current guidelines.

## Methods

We examined cardiac biomarker protocols from hospitals across the United States participating in Chest Pain Center Accreditation through the Society of Cardiovascular Patient Care (SCPC). In 2016, SCPC merged with the American College of Cardiology and now is referred to as the American College of Cardiology Accreditation Services. We defined a biomarker protocol as the chosen type of biomarker(s), time intervals between biomarker draws, number of laboratory draws, and diagnostic cut points used by a hospital in patients presenting for evaluation of suspected AMI. Hospitals undergoing Chest Pain Center Accreditation through the SCPC complete a survey addressing biomarker protocols:

1. Biomarker(s) used in their protocol to evaluate for suspected AMI.
2. Time interval between draws for each biomarker(s).
3. Assays and diagnostic cut points (with indeterminate range if applicable) used.

The SCPC accreditation is valid for 3 years, and criteria for accreditation are updated in 3‐year periods. Accreditation required serial cTn sampling out to at least 6 hours for myocardial infarction exclusion; otherwise, no specific marker protocol was recommended. Results from 824 hospitals were obtained during accreditation periods January 2009 to December 2011 and January 2012 to December 2014. The decision to restrict assessment to these time periods was done to reflect contemporary practice, and detailed biomarker information was not previously collected by SCPC. A hospital could have maintained or changed any aspect of biomarker protocol, assays, or cut points at any time.

First, we identified which biomarker(s) were used at each hospital for their suspected AMI evaluation protocol and described temporal changes in their usage. Each individual hospital protocol was sorted by year and categorized into 4 groups: cTn only; cTn+CK−MB; cTn+myoglobin; and cTn+CK−MB+myoglobin. Hospitals reporting from both accreditation periods were internally compared.

Second, we assessed the timing and number of biomarker draws used by an institution to exclude AMI. The baseline sample was considered hour 0, whereas the final biomarker draw was used to calculate the rule‐out time. Rule‐out periods were separated into 4 time intervals (≤3, 4–6, 7–9, and >10 hours), representing the most commonly used time intervals as well as reflecting rule‐out periods from older guidelines. Protocols were excluded from analysis if a biomarker was indicated as being used but the institution did not report biomarker sample timing.

Third, we identified the diagnostic cut‐point values used for cTn and assessed for changes over time. Reported cut points and assays were assessed against published reference values, and institutions were categorized as using cut points at the 99th percentile, at the 10% coefficient of variation level, or at a value above or below the 99th percentile.^7^ Protocols were excluded from analysis if either the cut point or the assay was not reported. Institutions that implemented 2 decision points (an indeterminate cTn range) were documented, and the primary decision point to define AMI was categorized into 1 of the 4 cut‐point groups defined above. The cTn cut‐point category and use of 2 decision points were assessed for temporal changes during the accreditation period.

### Statistical Analysis

Annual selection in biomarker groups and troponin cut points were analyzed. These categorical variables were reported as frequencies, and Fisher's exact test was used to determine statistical significance of the trend line from the study period. The most recent biomarker protocol was incorporated in the analysis for hospitals reporting protocol data from more than 1 accreditation period.

## Results

**Figure 1:**
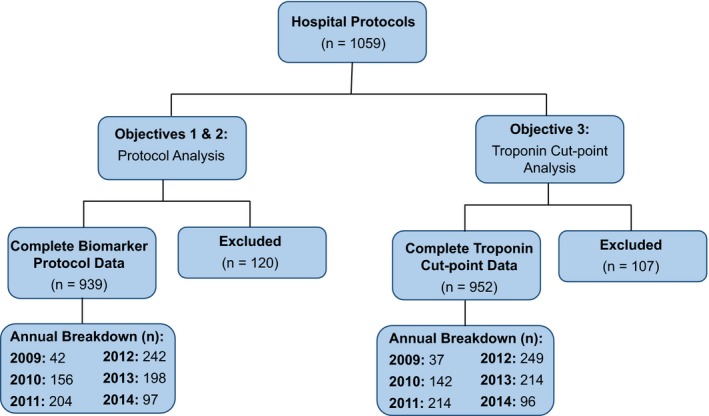
Study flow diagram. From the 2 accreditation periods (2009–2011, 2012–2014), data from 1059 biomarker protocols were assessed. There were 939 protocols (from 749 hospitals) that contained complete data of biomarker type, timing, and draw frequency. Nine hundred fifty‐two protocols (from 762 hospitals) contained chosen troponin cut point and reference assay. Protocols with absence of any component in reported data set were excluded from their respective analysis.

Data were available from 824 US institutions, of which 235 had data from both accreditation periods. Cumulatively, this resulted in information from 1059 protocols, from 2009 to 2014. Of these, 939 reported comprehensive information regarding biomarker type and the timing and frequency of draws (120 excluded), and 952 reported data for cTn cut‐points analysis (107 excluded; Figure 1).

### Biomarker Protocols/Combinations

**Figure 2:**
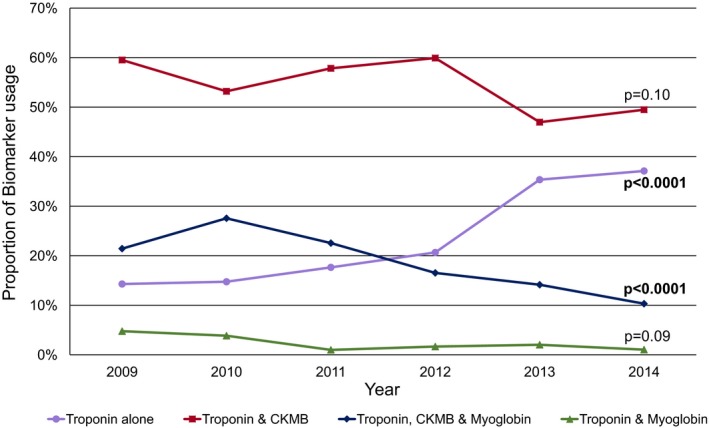
Trends in biomarker protocols. Annual utilized proportion of the 4 biomarker protocols (troponin alone; troponin and myoglobin; troponin and CK‐MB; and troponin, CK‐MB, and myoglobin). CK indicates creatine kinase.

The most commonly used biomarker protocol was the combination of CK‐MB and cTn, which decreased by ≈20% over the time period of the study (60% of hospitals in 2009, decreasing to 49% in 2014). A similar declining trend was observed for the combined use of CK‐MB, myoglobin, and cTn, which decreased by over 50% (21% in 2009, falling to 10% in 2014; *P*<0.0001). In contrast, use of cTn‐only protocols (24%) increased more than 2.6‐fold from 14% to 37% during the same time interval (*P*<0.0001). The combination of cTn and myoglobin (without CK‐MB) was infrequently used (Figure 2).

Comparing hospitals with data from both accreditation periods, 55% maintained the same biomarker combination, 35% changed their protocol to include fewer biomarkers (74% of which changed to a cTn‐only strategy), and 10% added an additional biomarker to their protocol. Of the protocols using fewer biomarkers in the second accreditation period, 60% changed which cTn assay that was used.

### Time Intervals for Evaluation of Suspected Acute Coronary Syndromes

**Figure 3:**
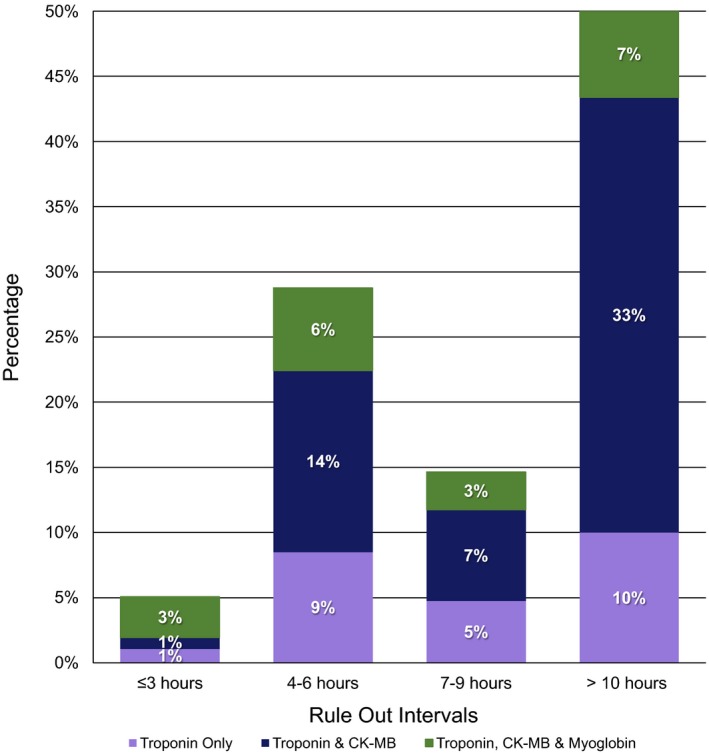
Frequency of AMI evaluation time intervals. Relative use of the 4 AMI evaluation time intervals throughout the 2009 to 2014 study period. The frequency of use of each biomarker combination within a time interval is shown above. AMI indicates acute myocardial infarction; CK, creatine kinase.

The most common interval was >10 hours (50%), followed by 4 to 6 hours (30%; Figure 3). The ≤3‐hour interval was used by only 5% of institutions, of which 62% used a triple biomarker strategy (cTn, CK‐MB, and myoglobin). When myoglobin was incorporated into a protocol, evaluation intervals were ≤3 hours (25%), 4 to 6 hours (32%), 7 to 9 hours (13%), and >10 hours (30%). There was no statistically significant temporal change in any of the time intervals during the study period.

### Individual Biomarker Temporal Sampling

cTn was incorporated into all protocols, whether alone or in combination with other biomarkers. Assays for cTnI were used in 90% of institutions and the remaining 10% used cTnT. Among these, 52 different cTn sampling intervals were identified. The 3 most common sampling intervals, accounting for 64% of the protocols, were 0, 6, and 12 (39%); 0, 3, and 6 (13%); and 0, 4, and 8 (12%) hours. Most protocols (90%) that used cTn collected samples at 3 time points. Of hospitals included in both accreditation periods, 38% changed their biomarker sampling time with 22% of these checking the second or third cTn at an earlier time point.

A total of 688 protocols (73.3%) incorporated CK‐MB, either in combination with cTn (54.5%) or both cTn and myoglobin (18.7%). Of the 43 different time intervals at which CK‐MB was drawn, the 3 most common were 0, 6, and 12 (43%); 0, 3, and 6 (12%); and 0, 8, and 16 (9%) hours. Most protocols (91%) that used CK‐MB specified 3 samples.

There were 195 protocols (20.7%) that used myoglobin in combination with cTn or CK‐MB and cTn. Of these, there were 29 different time intervals at which myoglobin was drawn. The 3 most common were 0, 6, and 12 (24%); 0, 3, and 6 (12%), and 0 and 2 (12%) hours. Overall, 79% of hospitals that incorporated myoglobin into a protocol drew 3 samples.

### Troponin Cut Points

**Figure 4:**
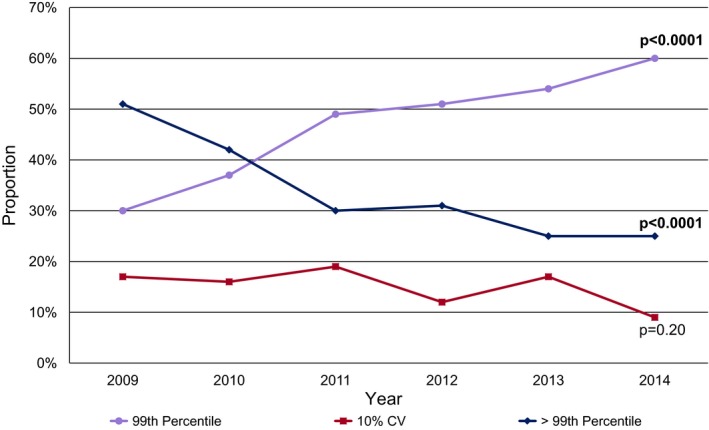
Trends in cardiac troponin cut points. Annual proportions of 99th percentile, 10% CV, and >99th percentile (referring to any chosen cut point greater than the 99th percentile) as cardiac troponin cut points. There a declining trend of use of cut‐point values above the 99th percentile and an increasing use of the 99th percentile cut point. CV indicates coefficient of variation.

Cumulatively from 2009 to 2014, the most commonly used cTn cut point was the 99th percentile (49%). Use of the 99th percentile as a cut point steadily increased from 30% in 2009 to 60% in 2014 (*P*<0.0001; Figure 4). Use of a value greater than the 99th percentile but that was not the 10% coefficient of variation level was the next most commonly used (31%). Use of a value greater than the 99th percentile decreased 2‐fold, from 51% in 2009 to 25% in 2014. Overall, use of a 10% coefficient of variation cut point was reported for 15% of the protocols and declined over time. The use of 2 decision points, which included an indeterminate cTn range, declined from 62% in 2009 to 44% in 2014 with a cumulative mean from 2009 to 2014 of 49%.

Subgroup analysis of the 10% of institutions using cTnT assays revealed a higher prevalence of cut points greater than 99th percentile (45%) with 20% using the 99th percentile.

## Discussion

There has been substantial evolution in the use of cardiac biomarkers for diagnosis of AMI.^2^, ^3^, ^8^, ^9^ To shorten the time to exclusion of AMI, different combinations of markers have been utilized, including various combinations of cTn, CK‐MB, and myoglobin. Because of its superior sensitivity and diagnostic accuracy, cTn is considered the gold‐standard biomarker for risk stratification and diagnosis of AMI.^2^, ^3^, ^8^, ^9^, ^10^, ^11^, ^12^ The recommendation to use cTn as a sole biomarker has remained consistent through the first, second, and third universal definitions of myocardial infarction published in 2000, 2007, and 2012, respectively. The 99th percentile is also recommended by the current American College of Cardiology/American Heart Association guidelines from 2014.^2^, ^3^, ^8^, ^9^ Despite the recommendations of these groups, we found that a minority of hospitals currently use a cTn‐only strategy, although the proportion has increased substantially. Our data are consistent with the findings from the CARMAGUE study, which surveyed 300 European hospitals and reported that 31% of laboratories used cTn as the sole marker for diagnosis of AMI.^13^ Thus, there appears to be a world‐wide issue with adaptation of recommended guidelines for use of cTn that may have an important impact on public health.

Before the development of more‐sensitive cTn assays, varying combinations of cTn, CK‐MB, and myoglobin have been used to improve early diagnostic sensitivity and reduce the time to rule‐out AMI. However, contemporary cTn assays have high early diagnostic accuracy, such that use of other markers adds cost^14^, ^15^, ^16^ without providing additional diagnostic utility.^17^, ^18^ This led the 2014 American College of Cardiology/American Heart Association non‐ST‐segment elevation myocardial infarction guidelines to indicate that there is no benefit (class III recommendation) of using myoglobin and CK‐MB, supported with a grade A level of evidence.^2^ Although we found marker combinations frequently used, there was decrease over time, concomitant with an increased use of a cTn‐only strategy, consistent with current guidelines and recommendations.

In addition to biomarker selection, timing of draws in the evaluation of suspected AMI has evolved, because increased sensitivity of cTn assays has shortened intervals required to exclude AMI. The 2007 second redefinition of myocardial infarction recommended sampling on presentation followed by 6 to 9 hours, which has been shortened to 3 to 6 hours in the more‐recent recommendations.^2^, ^3^, ^8^ We found that only one third of hospitals’ protocols excluded AMI within 6 hours. This may, in part, relate to delayed uptake in guidelines because our data were collected from hospitals undergoing accreditation during 2009 to 2014. Because of the time lags in the accreditation process, there would have been limited time for hospitals to modify their local protocols.

Adoption of the 99th percentile as a single cut point as recommended as a Laboratory Medicine Best Practice^6^ holds significant diagnostic and prognostic implications in the evaluation of AMI, given that it increases the diagnosis of AMI and identifies patients at greater risk for both readmission and recurrent AMI in comparison with higher cut points.^4^, ^5^ We found wide variation in the choice of diagnostic cut points by institution, with cumulatively less than half (49%) using the 99th percentile, although use did increase over time. Others have also found a wide variation in decision cut‐point values for cTn despite recommendations to use the 99th percentile for diagnosis.^13^, ^19^ Although not a recommended practice, we found a significant proportion of hospitals (49%) still using 2 decision points to define an indeterminate troponin range.^3^ These findings were similar to another study,^19^ in which 52% of the 649 hospitals reported using an indeterminate range.

Our study has some limitations. Data were self‐reported and because of limitations in data reporting, several institutions were excluded from the analysis. In addition, data were obtained from hospitals undergoing SCPC chest pain accreditation and may not be reflective of practice at all US hospitals. Thus, our data may overestimate guideline compliance and reflective of hospitals that have a commitment to quality improvement. Although biased by self‐selection, the hospital database represents a diverse and significant proportion of US hospitals from 48 states, including both academic and community, rural, and urban hospitals. Institutions may have changed assay platforms after submitting their data; if this occurred, it would not be reflected in our results.

## Conclusion

Multiple biomarker protocols are used in US hospitals for evaluation of patients with possible AMI. Use of cTn‐only protocols and using the 99th percentile cut point for cTn as recommended by current guidelines increased over time; however, substantial variation remains in diagnostic protocols and compliance with current guidelines. Our results have important implications for the diagnosis and treatment of patients with AMI.

## Disclosures

Dr Kontos is a consultant for Roche, AstraZeneca, and Provencio. Dr Newby is a consultant for BioKier, Metanomics, Merck, Roche Diagnostics, and Philips Healthcare; is a researcher for Metanomics, Sanofi, GlaxoSmithKline, and Verily (formerly Google Life Sciences); is on the data and safety monitoring board of DemeRx; is on the advisory board of MedScape/theHeart.org; and has received research funding from the National Institutes of Health and the Patient‐Centered Outcomes Research Institute; and has received honoraria from *JACC: Basic to Translational Science* and the *Journal of the American Heart Association*. Dr Christenson is a consultant for Roche Diagnostics, Philips, and Siemens Healthcare and has research funding from Roche, Siemens, Mitsubushi, Abbott, Beckman Coulter, Alere, and Ortho Diagnostics. Dr Peacock has obtained research grants from Abbott, Alere, Banyan, Cardiorentis, Janssen, Portola, Roche, and ZS Pharma; served as a consultant for Alere, Beckman, Boehringer Ingelheim, Cardiorentis, Instrument Labs, Janssen, Phillips, Prevencio, Singulex, The Medicine's Company, and ZS Pharma; and has ownership interests in Comprehensive Research Associates, LLC, and Emergencies in Medicine, LLC. Dr McCord is a consultant for and receives research funding from Roche Diagnostics and Siemens Diagnostics. All other authors have reported that they have no relationships relevant to the contents of this article to disclose.
